# Oligoadenylate synthase-like (OASL) proteins: dual functions and associations with diseases

**DOI:** 10.1038/emm.2014.110

**Published:** 2015-03-06

**Authors:** Un Yung Choi, Ji-Seon Kang, Yune Sahng Hwang, Young-Joon Kim

**Affiliations:** 1 Department of Biochemistry, College of Life Science and Technology, Yonsei University, Seoul, Korea; 2 Department of Integrated Omics for Biomedical Science, Yonsei University, Seoul, Korea

## Abstract

The study of antiviral pathways to reveal methods for the effective response and clearance of virus is closely related to understanding interferon (IFN) signaling and its downstream target genes, IFN-stimulated genes. One of the key antiviral factors induced by IFNs, 2′-5′ oligoadenylate synthase (OAS), is a well-known molecule that regulates the early phase of viral infection by degrading viral RNA in combination with RNase L, resulting in the inhibition of viral replication. In this review, we describe OAS family proteins from a different point of view from that of previous reviews. We discuss not only RNase L-dependent (canonical) and -independent (noncanonical) pathways but also the possibility of the OAS family members as biomarkers for various diseases and clues to non-immunological functions based on recent studies. In particular, we focus on OASL, a member of the OAS family that is relatively less well understood than the other members. We will explain its anti- and pro-viral dual roles as well as the diseases related to single-nucleotide polymorphisms in the corresponding gene.

## Introduction

**Figure 1:**
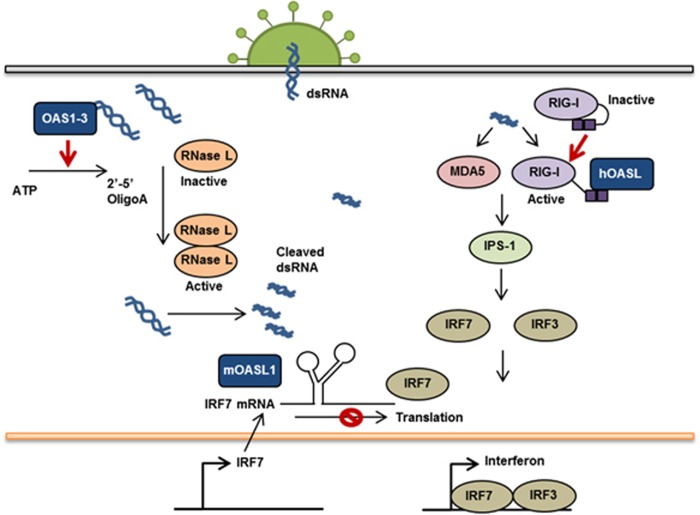
The OAS family in antiviral pathways. Following virus infection of a host cell, viral dsRNA stimulates OAS1, OAS2 and OAS3 and leads to the synthesis of 2′-5′ oligoA and RNase L activation. Activated RNase L cleaves viral and cellular RNA. MDA5 and RIG-I detect cleaved viral RNA, thereby promoting the activation of IRF3 and IRF7. The hOASL binds to RIG-I and enhances the sensitivity of RIG-I signaling through the UBL domain, whereas the mOASL1 binds to IRF7 mRNA and inhibits the translation of IRF7.

To counter virus infection, the immune system produces antiviral cytokines. Interferon (IFN) is the most powerful antiviral cytokine, and it induces IFN-stimulated genes that mediate antiviral effector functions. Among the proteins induced by IFN, the oligoadenylate synthase (OAS) proteins have been identified as enzymes that sense exogenous nucleic acid and initiate antiviral pathways. The OAS family proteins belong to a template-independent nucleotidyltransferase family. The oligomerized OAS enzyme generates the 2′-5′-linked oligoadenylate (2-5A) activating endoribonuclease, RNase L, which degrades cellular and viral RNA. In this way, RNase L contributes to the control of the early spread of a virus by degrading the viral RNA and activating cytoplasmic pattern-recognition receptors, including RIG-I and MDA-5 (Figure 1). Although the antiviral activity of the OAS family and 2-5A-RNase L is well characterized,^1, 2, 3^ it was reported recently that not all of the OAS antiviral function is mediated by the RNase L-dependent pathway.^4, 5^ This indicates that the OAS family proteins may be involved in pathways for regulating viral infection other than the pathway involving RNase L activation.

The OAS family proteins consist of OAS1, OAS2, OAS3 and OAS-like protein (OASL).^6^ The OAS1-3 proteins have significant homology to each other and only differ in the number of OAS units. OAS1, OAS2 and OAS3 contain one, two and three OAS units, respectively. The genes encoding the OAS proteins are clustered on chromosome 12 (in the 12q24.1 region) in humans. Human OAS1 (hOAS1) has two spliced forms that produce proteins of 40 and 46 kDa, each containing a distinct C-terminal sequence.^7^ Three more alternative splice forms of *Oas1* are generated by single-nucleotide polymorphisms (SNPs).^8^ There are two isoforms of OAS2 (p69 and p71), and one 100 kDa OAS3 form.^7^ Two isoforms of hOASL OASLa (p59) and OASLb (p30) are coded by a gene located on chromosome 12 (in the 12q24.2 region), but OASLa is the dominant isoform; OASLb has a C-terminal truncation. Recently, another OASL isoform, OASLd, which is strongly induced by IFNγ, was discovered.^9^ In the mouse genome, eight *Oas1* genes and one gene each for OAS2 and OAS3 exist on chromosome 8.^10^ Mice have two OASL genes (*Oasl1* and *Oasl2*) on chromosome 5.^11^ Studies using genetically modified mouse models for the OAS genes may allow us to gain insights into the human OAS family functions, because the exon/intron structures of all the genes are conserved between humans and mice.^10^ In this article, we review the immune-related and other cellular functions of OAS and the dual role of OASL. We then discuss SNPs and the differential expression of OAS in various diseases.

## RNase L-dependent and -independent antiviral functions of OAS1, OAS2 and OAS3

The canonical OAS/RNase L pathway has been intensively studied, and the results have been well documented in several reviews.^6, 12^ The ability to activate RNase L of the OAS family is dependent on the OAS oligomerization unit and the processivity of synthesizing dimeric or tri-tetrameric 2-5A. The CFK motif in the OAS domain, which is required for tetramerization of OAS and affects the synthesis of effective 2-5A, is only conserved between OAS1 and OAS2.^13^ Originally, it was believed that only OAS1 and OAS2, and not OAS3, contributed to RNase L-dependent antiviral activity.^13, 14^ This idea has been revised by studies of mouse OAS1b. The observation that point mutations of mOas1b increased the susceptibility of mice to West Nile Virus (WNV) indicated that the OAS/RNase L system is required for virus restriction.^5, 15^ Recent experiments with each of the isoforms of the OAS family overexpressed cell revealed that OAS3 shows RNase L-dependent antiviral activity against dengue virus.^16^ Furthermore, purified OAS3 can produce long enough 2-5A to activate RNase L.^17^ In an antiviral response to hepatitis C virus, overexpressed OAS1 p46 and OAS3 p100 show antiviral activity by mediating the RNase L-dependent pathway.^18^ OAS3 synthesizes dimeric 2-5A that binds to RNase L with low affinity and produces 2-5A oligomers shorter than the tri-tetramer 2-5As produced by OAS1 and OAS2. However, OAS3 can be induced by smaller amounts of double-stranded RNA (dsRNA) than OAS1 and OAS2.^19^ The higher dsRNA-binding affinity of OAS3 to dsRNA might compensate for the lower binding affinity of dimeric 2-5A to RNase L.

Recent studies have focused on the OAS expression patterns after various virus infections and the polymorphisms associated with virus infection susceptibility. Dengue virus infection causes the early induction of OAS1, whereas OAS2 and OAS3 are upregulated later.^20, 21^ Studies of polymorphisms in the Oas genes have emphasized the importance of all the three Oas genes during dengue virus infection.^22^ In the case of Chikungunya virus (CHIKV), OAS3 shows a strong correlation with resistance to CHIKV infection. Mutation of Oas3 caused less efficient inhibition of CHIKV, regardless of RNase L activation.^23^ CHIKV with the E2-E116K substitution escaped the antiviral action of OAS3 and replicated more successfully than the wild-type virus by reducing OAS3 expression.^24, 25^ Therefore, OAS3 appears to be required for the restriction of specific viruses, independent of RNase L.

## The OASL: an antiviral or pro-viral protein?

OASL contains one OAS domain, two ubiquitin-like repeats and a CCY motif instead of CFK in the OAS unit that is required for oligomerization. Thus the hOASL lacks the 2′-5′-linked OAS activity that is one of the hallmarks of OAS.^26^ Expression of OASL is also regulated differently from OAS.^27^ Whereas mOASL1 is mainly induced by IFNa, mOASL2 is upregulated by both type I and type II IFN.^28^ Unlike hOASL, mOASL2 maintains OAS activity and requires dsRNA as an inducer. The amino-acid sequence of mOASL1 is more similar to human OASL than mOASL2 is (mouse OASL1, 74% mouse OASL2, 49% identical with hOAL).^10^ Thus it is plausible that mOASL1 is the functional counterpart and ortholog of hOASL. It has been suggested that mOASL2 is an intermediate between OAS and OASL that arose during evolution.^11^

The role of OASL was identified later than that of OAS, but the recently identified functions seem to be critical to the antiviral innate and adaptive immune responses. These facts make further studies of OASL valuable. Because of its OAS-like qualities (activation by IFN and binding to dsRNA) and inactive nucleotidyltransferase domain, OASL has been assumed to interfere with the 2-5A and RNase L pathway by competing with OAS.^29, 30^ An SNP study of the response to IFN therapy for chronic hepatitis C suggested that OASL might negatively regulate the antiviral function of OAS. An SNP that cause lower levels of OASL was observed to be associated with a sustained virological response after treatment.^31^ However, it has been demonstrated that hOASL possesses antiviral activity through the C-terminal ubiquitin-like domain. In Vero cells, hOASL expression increases the resistance against single-stranded RNA viruses, including picornavirus and encephalomyocarditis, but not against a DNA virus, herpes simplex virus 1.^32^ A recent study using an OASL deletion mutant produced by a transcription activator-like effector nucleases (TALEN) procedure found that hOASL suppresses the replication of vesicular stomatis virus by enhancing the RIG-I pathway (Figure 1). In addition, this study established that mOASL2 also has an antiviral activity, suggesting that mOASL2 is functionally comparable to hOASL.^33^ The function of mOASL1 is totally different from that of mOASL2 and hOASL. The mOASL1 protein inhibits the translation of IFN-regulating transcription factor 7, the main transcription factor for type I IFN, and suppresses the production of type I IFN during viral infection^34^ (Figure 1) through an interaction with the stem loop structure in the 5′-untranslated region of IFN-regulating transcription factor 7.^35^ Negative regulation of type I IFN production by mOASL1 causes viral persistence and represses T-cell function.^36^ Together, OASL proteins have dual functions that depend on various mechanisms and on the phase of the virus infection.

## The OAS family as a biomarker

Expression of the OAS family is upregulated in some diseases whether it is dependent or independent of IFN stimuli. In light of this, OAS can be a useful biomarker for various diseases in multiple stages. A specific biomarker is needed to select an adequate therapy for an individual and to monitor the effect of the therapy. The OAS level is strongly related to autoimmune diseases and chronic infections, including systemic lupus erythematosus, systemic sclerosis, rheumatoid arthritis and multiple sclerosis.^37, 38, 39, 40^ Appropriate biomarkers must express specific patterns depending on the condition of a disease. In the study of systemic lupus erythematosus, it was shown that OAS1 is upregulated, whereas expression of OASL was lower in systemic lupus erythematosus patients than in normal individuals.^41^ In primary human peripheral blood mononuclear cells isolated from systemic sclerosis patients, only the expression of OAS2 and OASL was higher than in the basal state and neither OAS1 nor OAS3 was induced.^37^ Patients with severe chronic obstructive pulmonary disease show high mortality. Exposure to influenza virus and cigarette smoke leads to a more serious stage of chronic obstructive pulmonary disease. In a mouse experiment, only OAS2 and OASL were synergistically induced by cigarette smoke and influenza virus, whereas OAS1 and OASL2 were not induced.^42^ OASL also can be used as a biomarker to predict rheumatoid arthritis patients' response to tocilizumab, the drug most commonly used to treat this disease. Expression of OASL differs significantly between nonresponders and responders.^43^ The expression pattern of the OAS family may offer useful information for therapy of autoimmune diseases and chronic infections and reveal different roles of each member of the OAS family in autoimmune disorders.

## SNPs in OASL-associated diseases

Several SNPs in the OAS family genes have been identified and associated with various diseases (Table 1). Consistent with the main role of OAS proteins, SNPs in OAS genes affect the susceptibility to viral infection. Most people who are infected with WNV of the Flaviviridae family show no symptoms, but a few people (<1%) progress to a severe clinical infection, West Nile fever.^44^ Genetic factors, age and environmental conditions may have essential roles in disease progression. Sequencing the OAS family exons in 33 patients with WNV infection showed that a SNP (rs3213545) of the OASL gene is associated with WNV infection.^45^ Although the rs3213545 SNP is synonymous in OASL exon2, it contains a splice enhancer site that is a minor allele ‘T', which generates a dominant-negative mutant form of OASL. A study using 331 WNV-infected patients showed that the OAS1 SNP rs10774671 has a significant link with WNV. These data support the conclusion that human OAS1 and OASL have antiviral roles against WNV. In contrast, the minor allele ‘T', the rs3213545 SNP, is significantly associated with a sustained virological response, an efficacy measure of hepatitis C virus treatment after IFN therapy;^31^ that is, diminished OASL activity confers an advantage for IFN treatment. Depending on the early response to the recovery phase, one of the dual roles of OASL can be addressed.

The role of OASL in diseases other than immune-related diseases is demonstrated by genome-wide association studies. The locus of the OASL gene on chromosome 12 was shown to affect multiple cardiovascular-related traits, especially ‘high or low' gamma glutamyltransferase, low-density lipoprotein and C-reactive protein. The study identified the rs3213545 SNP as a possible candidate associated with liver function and lipid constitution.^46^

## Non-immunological functions of the OAS family proteins

Although the major role of the OAS proteins is as immune regulators, there are some data showing that OAS proteins are involved in other cellular functions. The OAS family is associated with the regulation of apoptosis, one of the ways organisms react against virus infection in an effort to eliminate the virus-infected cells and a core mechanism for inhibiting tumorigenesis.^47, 48^ A derivative of the dimeric 2-5A-activating RNase L synthesized by OAS3 has been described as a new molecule for inhibiting breast cancer cell growth.^49^ OAS3 is also one of the genes in a breast tumor cell line (MCF7) that is highly expressed after daily exposure to radiation.^50^ In addition, the OAS/RNase L pathway is induced by BRCA1, a tumor suppressor of the breast and ovarian cancer, and it activates apoptosis of tumor cells.^51^ These observations implicate the OAS proteins in counteracting tumor progression.

Even though hOASL has a supposed nuclear localization signal (RKVKEKIRRTR) at the C-terminus, its nuclear function is not clear.^6^ However, there is some evidence that a nuclear function of OASL exists. A yeast two-hybrid system identified an interaction between OASL and methyl CpG-binding protein 1, which is a transcriptional repressor.^52^ In addition, it has been shown that OAS might also regulate nuclear events, including pre-mRNA splicing, a complicated process that requires a complex of RNA and proteins. OAS assembles the 60S spliceosome and is necessary for the first step of splicing.^53^

## Conclusion

The RNaseL-dependent immune regulation by the OAS family is widely known. However, various functions of each OAS family member and a newly defined mechanism independent of RNase L have been discovered recently. Because most of the studies focusing on the RNase L-independent functions of OAS employ *in vitro* methods, including genetic depletion, using an *in vivo* knockout mouse model would be useful for finding new mechanisms. Other important aspects are SNPs and the expression level changes of the OAS family during disease progression. Many SNPs in the OAS genes have been discovered to be associated with viral infections and autoimmune diseases. In addition, the expression levels of OAS family members depend on disease progression. Although we still lack a complete understanding of the roles of SNPs of the OAS family members in disease, their strong correlation with viral diseases will prove useful for identifying new treatment methods for infectious and autoimmune diseases.

## Figures and Tables

**Table 1:** SNPs in the OAS family

*Gene*	*SNP*	*Associated clinical phenotype*	*Location*	*Nucleotide change*	*SNP effect*
*Oas1*	rs10774671	WNV	Intron	G/A	Different splicing
		T1D			
	rs12307655	HPV	Intron	C/T	
	rs2660	SARS	Exon6	A/G	Arg/Gly substitution
		HCV			
					
*Oas2*	rs2010604	SVR	3′ UTR	G/C	
	rs1293762	SVR	Intron	T/G	
		TBEV			
	rs15895	TBEV	3′ UTR/exon11	A/G	Early stop codon
	rs1732778	TBEV	3′ UTR	G/A	
	rs718802	HPV	3′ UTR	C/A	
					
*Oas3*	rs2285932	TBEV	Exon6	T/C	Synonymous
	rs2072136	HBV	Exon2	C/G	Synonymous
		TBEV			
	rs12302655	HPV	5′ UTR	G/A	
					
*Oasl*	rs1169279	SVR	3′ UTR	A/G	
	rs3213545	SVR	Exon2	C/T	Synonymous
		WNV			
		MCRT			
	rs2859398	SVR	Promoter	C/T	

Abbreviations: HCV, hepatitis C virus;^54^ HPV, human papillomavirus;^55^ MCRT, multiple cardiovascular-related traits; OAS, oligoadenylate synthase; SARS, severe acute respiratory syndrome;^56^ SNP, single-nucleotide polymorphism; SVR, sustained virologic response; TBEV, tick-borne encephalitis virus;^58^ T1D, type I diabetes;^57^ UTR, untranslated region; WNV, West Nile Virus.
